# Differential analysis of histopathological and genetic markers of cancer aggressiveness, and survival difference in EBV-positive and EBV-negative prostate carcinoma

**DOI:** 10.1038/s41598-024-60538-0

**Published:** 2024-05-05

**Authors:** Khalid Ahmed, Alisalman Sheikh, Saira Fatima, Tahira Ghulam, Ghulam Haider, Farhat Abbas, Antonio Sarria-Santamera, Kulsoom Ghias, Nouman Mughal, Syed Hani Abidi

**Affiliations:** 1 Department of Biological and Biomedical Sciences, Aga Khan University, Karachi, Pakistan; 2 Department of Pathology and Laboratory Medicine, Aga Khan University, Karachi, Pakistan; 3 Department of Surgery, Aga Khan University, Karachi, Pakistan; 4 Department of Biomedical Sciences, Nazarbayev University School of Medicine, Astana, Kazakhstan

**Keywords:** EBV, LMP1, Prostate carcinoma, Gleason scores, Tumor grade, Survival analysis, EMT markers, Oncogenes, Gene expression, Cancer, Microbiology

## Abstract

Several studies have shown an association between prostate carcinoma (PCa) and Epstein-Barr virus (EBV); however, none of the studies so far have identified the histopathological and genetic markers of cancer aggressiveness associated with EBV in PCa tissues. In this study, we used previously characterized EBV-PCR-positive (n = 39) and EBV-negative (n = 60) PCa tissues to perform an IHC-based assessment of key histopathological and molecular markers of PCa aggressiveness (EMT markers, AR expression, perineural invasion, and lymphocytic infiltration characterization). Additionally, we investigated the differential expression of key oncogenes, EMT-associated genes, and PCa-specific oncomiRs, in EBV-positive and -negative tissues, using the qPCR array. Finally, survival benefit analysis was also performed in EBV-positive and EBV-negative PCa patients. The EBV-positive PCa exhibited a higher percentage (80%) of perineural invasion (PNI) compared to EBV-negative PCa (67.3%) samples. Similarly, a higher lymphocytic infiltration was observed in EBV-LMP1-positive PCa samples. The subset characterization of T and B cell lymphocytic infiltration showed a trend of higher intratumoral and tumor stromal lymphocytic infiltration in EBV-negative tissues compared with EBV-positive tissues. The logistic regression analysis showed that EBV-positive status was associated with decreased odds (OR = 0.07; *p*-value < 0.019) of CD3 intratumoral lymphocytic infiltration in PCa tissues. The analysis of IHC-based expression patterns of EMT markers showed comparable expression of all EMT markers, except vimentin, which showed higher expression in EBV-positive PCa tissues compared to EBV-negative PCa tissues. Furthermore, gene expression analysis showed a statistically significant difference (*p* < 0.05) in the expression of *CDH1, AR, CHEK-2, CDKN-1B*, and *CDC-20* and oncomiRs *miR-126, miR-152-3p, miR-452, miR-145-3p, miR-196a, miR-183-3p*, and *miR-146b* in EBV-positive PCa tissues compared to EBV-negative PCa tissues. Overall, the survival proportion was comparable in both groups. The presence of EBV in the PCa tissues results in an increased expression of certain oncogenes, oncomiRs, and EMT marker (vimentin) and a decrease in CD3 ITL, which may be associated with the aggressive forms of PCa.

## Introduction

Despite being one of the most common carcinomas in men worldwide^1^, the infectious etiology and events related to the onset and progression of prostate carcinoma (PCa) remain poorly understood^2,3^. Several studies have shown an association between prostate carcinoma (PCa) and Epstein-Barr virus (EBV)^3–5^. Our recent report showed that EBV maintains a latency II/II-like profile in PCa tissues, and EBV-PCR-positive PCa tissues exhibited higher Gleason scores compared to EBV-negative PCa tissues^4^. However, it is not known whether the presence of EBV in PCa tissues is associated with histopathological and gene expression changes associated with cancer aggressiveness.

In the absence of mechanistic studies supporting the histopathological findings, it can be hypothesized that EBV may have an essential role in the PCa progression plausibly through its oncogenic proteins^6^. For example, LMP1, a major EBV oncoprotein, plays a critical role in the oncogenesis of EBV-driven malignancies and also serves as a surrogate marker for the detection of EBV in the cancer tissues^7–9^. It has been shown to drive epithelial-mesenchymal transition (EMT) in nasopharyngeal carcinoma^10,11^. In addition to EMT, androgen receptor signaling pathways also have a crucial role in the carcinogenesis of PCa, where androgen receptor (AR) downregulation leads to the acquisition of castration-resistant phenotype of PCa refractory to the conventional treatment, contributing toward the progression of PCa^12^. Previously, human herpesvirus-8 (HHV8) has been shown to cause AR downregulation, associated with PCa progression^13^, however, this is unknown whether EBV can also lead to AR downregulation and PCa progression.

Similarly, the alterations in the gene expression of *c-Myc, BCL-2, TP53, MDM-2, Rb, BRCA-1, BRCA-2, CDKN-1B, CDKN-1A*, and *CDKN-1B* have shown to be associated with PCa progression^14,15^, and EBV has been shown to modulate several of these genes in other cancer types^4,16^, however, the EBV-associated dysregulation of gene expression in PCa has not been explored. In addition, certain prostate-specific miRNAs (oncomiRs) reportedly regulate a wide array of prostatic functions, ranging from proliferation to apoptosis. Therefore, by modulating these functions, the oncomiRs might be driving the onset/progression of PCa^17,18^. Viruses, such as HPV, can cause dysregulation of certain oncomiRs, such as *miR-29b*, *miR-34a-5p*, and *miR-146a-5p*, which inhibits c-Myc in an E-6-dependent manner contributing to the progression of PCa^19^. Similarly, EBV-LMP-1 mediated overexpression of miR-146a in nasopharyngeal cells represses the interferon-responsive gene, resulting in proliferation and bypassing of immune surveillance^20,21^. In light of these observations, it is logical to hypothesize that EBV-mediated dysregulation of prostatic oncomiRs might play an important role in the progression of PCa.

As a sequel to our previous study^22^, here we assessed the histopathological differences in markers of PCa progression, including perineural invasion (PNI), T and B lymphocytic infiltration, and IHC-based expression of EMT marker proteins (E-cadherin, N-cadherin, and vimentin), as well as AR in EBV-positive and EBV-negative PCa tissues. This was followed by the assessment of differential mRNA expression analysis of selected genes and oncomiRs significantly implicated in oncogenesis and EMT in prostate cancer^15,23^.

## Materials and methods

### Samples collection

This study was based on 99 previously characterized FFPE prostate carcinoma (PCa) tissues, belonging to prostatic adenocarcinoma of acinar type^4^. Since these samples were sent to the Aga Khan University histopathology laboratory in 2019, only for histopathological assessment, the relevant clinical parameters were not available to us. The study was approved by the Aga Khan University Ethics Review Committee (AKU-ERC #: 2021-1460-18525), and samples were acquired after obtaining informed consent from all subjects.

In our previous study on these PCa tissues, using PCR assay based on 14 EBV genes, namely *EBNA-3B, EBNA-3A, EBNA-2, EBNA-1, LMP-2A, LMP-2, LMP-1, EBNA-LP, EBNA-3C, EBNA-2B, EBER-2, EBER-1, BZLF-1,* and *BHRF-1*, we detected EBV in 39/99 FFPE prostate carcinoma (PCa) tissues^4^. Based on the PCR status, 39 samples were characterized as EBV-positive and 60 samples were characterized as EBV-negative^4^. Subsequently, we performed differential analysis of key histopathological features of EBV-positive (n = 39) and EBV-negative (n = 60) PCa tissues.

### Immunohistochemical detection of EBV LMP1 protein in prostate carcinoma tissues

We also adopted IHC-based EBV LMP-1 expression as an approach to further confirm the EBV positivity status. For this analysis, 31 out of 39 EBV PCR-positive PCa samples were used for immunohistochemical expression analysis of EBV LMP1 oncoprotein, while eight samples were dropped due to insufficient tissue. The formalin-fixed tissue sections were deparaffinized and rehydrated. Antigen retrieval was enhanced by revealing epitopes with the use of citrate buffer at pH 9.0. Subsequently, the sections were stained with a mouse antibody against EBV LMP1 (CS1-4) (Dako, Agilent Technologies Denmark). The detection of LMP1 was carried out through staining with horseradish peroxidase-labeled anti-mouse secondary antibody, while Diaminobenzidine (Dako DAB) was used as a substrate chromogen, and hematoxylin was used as a counterstain. The slides were further processed using Autostainer Link 48 (Dako Agilent) following the manufacturer's instructions. The slides showing either membranous or cytoplasmic staining (brown color) of tumor cells in the specimens were considered positive for LMP1^24,25^. The slides were scored using the following criteria described by Mao et al.^25^ score 4 = 81–100% LMP1 positivity, score 3 = 51–80% LMP1 positivity, score 2 = 11–50% LMP1 positivity, score 1 = 1–10% LMP1 positivity in the cells. Furthermore, the intensity of immunostaining was scored 0 for negative, while 1, 2, and 3 for weak, moderate, and strong immunostaining, respectively.

### Assessment of perineural invasion in EBV-positive and EBV-negative PCa tissues

The EBV-positive (n = 39) and EBV-negative (n = 60) PCa tissues were graded by an experienced histopathologist using the WHO 2016/ ISUP 2014-based prostate cancer grading system^26^, and Gleason scores and perineural invasion status of the tissues was documented^4^. Since Gleason scores comprise Gleason major (primary pattern), Gleason minor (secondary pattern), and Gleason total (combination of primary and secondary patterns) with assigned values of 5 for least differentiated to 1 for most differentiated in each of these categories^26,27^, therefore, the mean scores of Gleason major, Gleason minor, and Gleason total scores were obtained in both EBV-positive and EBV-negative groups, and those mean scores were subsequently used to correlate with the presence or absence of PNI using Spearman correlation analysis using GraphPad Prism 8.4 software. This correlation analysis between Gleason scores and PNI is important because, in our previous study, we have shown that EBV-positive PCa tissues exhibited higher Gleason total and Gleason major scores compared to EBV-negative PCa tissues^4^.

### Immunohistochemical characterization of T and B cell lymphocytic infiltration in EBV-positive and EBV-negative PCa tissues

To characterize the presence of lymphocytic infiltration, B (CD20+) and T (CD3+) cell characterization in the EBV-positive (n = 39) and EBV-negative (n = 60) PCa tissue, criteria from the International Immuno-Oncology Biomarker Working Group^22,28^ were used with modifications. On light microscopy, the Hematoxylin and Eosin-stained PCa tissues were scanned for the presence of lymphocytes and categorized into intratumoral and tumor stromal lymphocytic infiltration in the specimens. The intensity of lymphocytic infiltration was rated using a scale of 0 to + 3, with 0 indicating no lymphocytes observed, and + 1, + 2, and + 3 indicating the presence of 1–15, 16–25, and > 25 lymphocytes, respectively. A frequency distribution graph was obtained to show the distribution of lymphocytes in EBV-positive and EBV-negative PCa tissues using GraphPad Prism 8.4.

To characterize the presence of T and B lymphocytic infiltration in the tissues, the formalin-fixed tissue sections were deparaffinized and rehydrated. Antigen retrieval was enhanced by revealing epitopes using citrate buffer at pH 9.0. Subsequently, the sections were stained with a ready-to-use FLEX polyclonal rabbit anti-human CD3 antibody (IR503, Dako) and a ready-to-use FLEX monoclonal mouse anti-human CD20cy Clone L26 (IR604, Dako, Agilent Technologies Denmark). The detection of CD3 and CD20 was carried out through staining with horseradish peroxidase-labeled anti-mouse secondary antibody, while diaminobenzidine (Dako DAB) was used as a substrate chromogen, and hematoxylin was used as a counterstain. The slides were further processed using Autostainer Link 48 (Dako Agilent) following the manufacturer’s instructions.

In the next step, logistic regression analysis was performed to examine the influence of EBV status on different variables such as Gleason scores, lymphocytic infiltration (ITL), PNI, PGG, etc. The analyses were performed using IBM SPSS v22 software.

### RNA extraction, cDNA conversion, and qPCR-based differential mRNA expression analysis of selected oncomiRs and genes associated with oncogenesis and EMT in EBV-positive and EBV-negative PCa tissues

For gene expression analysis, the following genes were selected: *c-MYC, AURKB, CDC20, EP300, MDM2, TP53, RB1, CHEK2, CDKN-1B, CDKN2A, BRCA1, BRCA2, RAD51, CDK2, CDKN1A, RAD9A, MRE11, ATR, BCL2, KLK3, AR, CDH1, CDH2, VIM, SNAI2, SNAI1, TWIST1, and ZEB1* (Table S2). These genes have been implicated in prostate carcinogenesis and EMT, and are important markers of progression and aggression in prostate cancer^15,29^. Additionally, the following PCa-specific oncomiRs^30^ were also tested: *miR-126, miR-302, miR-16-5p, miR-205, miR-100-5p, miR-183-3p, miR-183-5p, miR-152-3p, miR-152-5p, miR-146b, miR-200b-3p, miR-196a, miR-34a-5p, miR-375, miR-145-3p, miR-145-5p, miR-634, miR-181, miR-29b, miR-146a-3p, miR-21-5p, miR-101, miR-106a-5p, miR-452* and *miR-182-5p *(Table S3).

Based on the EBV positivity in PCa samples (39/99), the sample size for the gene expression analysis was calculated using an online calculator (ClinCal)^31^ with EBV-positive incidence in PCa tissues at 39% and a type I error (α) at 5% and Power (1- β; β = type II error) at 80%. The sample size based on these parameters was calculated to be 15 for both groups. However, for gene expression analysis we used an equal number, in a ratio of 1:1, of EBV-positive (n = 20) and EBV-negative (n = 20) PCa tissues, higher than the calculated sample size. In the first step, we ensured that the representative samples (in terms of histopathological grading and expression, Gleason scores, etc.) from EBV-positive and EBV-negative samples were included. The final samples were randomly selected from each group to avoid bias.

In the first step, RNA was extracted using the standard TRIzol-chloroform method, followed by cDNA synthesis. 2 μl of cDNA sample was combined with a mixture containing 4 μl of BlasTaq™ (2X) qPCR master mix (ABM, Canada, Cat. No. G891), forward and reverse gene-specific primers (Table S2) (Macrogen, USA) and nuclease-free water to a final reaction volume of up to 10 μl in a 0.2 ml tube (Bio-Rad Laboratories, USA. Cat. No. TLS0851). The prepared reactions were subjected to the following thermal cycling conditions using Bio-Rad 1000 thermal cycler CFX96 (Bio-Rad laboratories, USA): initial denaturation at 95 °C for 10 min, followed by 40 cycles of denaturation at 95 °C for 15 s, annealing of primers from 48 to 50 °C for 20 for oncomiRs and 60–65 °C for 1 min for all other genes. A melt curve analysis was set up between 55 and 95 °C with an increment of 0.5 °C every 5 s to plot the specificity of the products. Each sample was run in duplicates, while non-template controls were supplied with an additional 2 μl of nuclease-free water instead of a cDNA template. The expression of each gene was calculated using the ∆CT method, while fold-change was calculated using the 2^−∆∆CT^ method^32^. For normalization, *RNA-U6* was used as the housekeeping gene in the case of the oncomiRs, while β-actin was used as the housekeeping gene for all other genes. Furthermore, since the data was found to be normally distributed (based on the D’Agostino-Pearson normality test), the statistically significant difference (< 0.05) in the expression of the tested gene in the two groups (EBV-positive versus EBV-negative) was compared using Unpaired T test with Welch’s correction.

### Gene ontology enrichment analysis

Gene enrichment/ontology analysis was used to examine the enrichment of target genes for differentially expressed miRNA within the PCa tissues. The target genes for each upregulated and downregulated miRNA were extracted using the miRDB database (https://mirdb.org/)^33^. These gene symbols were classified as upregulated and downregulated based on the query miRNA. Following this, these targets were analyzed for ontology and pathway enrichment using the ShinyGO v0.77 web-based tool (http://bioinformatics.sdstate.edu/go/)^34^ by selecting humans as identical species. For all the analyses, the top 20 pathways with a *p*-value < 0.05 were included.

### Immunohistochemical-based expression analysis of AR and EMT markers in EBV-positive and EBV-negative PCa tissues

EBV-positive and -negative FFPE PCa tissue were deparaffinized and rehydrated as per the standard protocol^35^, using xylene (cat# 116599-62-3, Sigma) and alcohol (cat# 64-17-5, Sigma). Subsequently, the slides were kept in preheated antigen retrieval buffer (50 × Tris/EDTA buffer) (EnVision Flex High pH, Dako, cat# K-80022) at pH 9.0 for 30 min (95–100 °C). The blocking agent (EnVision FLEX peroxidase blocking reagent, Dako) was applied for 30 min. The slides were flushed with 1X PBS three times, and the primary antibody was incubated for 1 h. The ready-to-use primary antibodies of vimentin Clone V9 (cat# IR630, DAKO) and E-Cadherin Clone NCH-38 (cat# IR059, DAKO) were used. For N-cadherin, N-Cadherin Clone 3B9 with a dilution of 1:50 (Cat# 33-3900, ThermoFisher Scientific) and androgen Receptor Clone AR441 with a dilution of 1:50 (cat# M3562, DAKO) were used. The dilution of N-Cadherin and AR antibodies was carried out using the Dako REAL antibody diluent (cat# S2022). Only for the AR antibody, the slide was treated with 0.1% Triton X (cat# 9036-19-5, Sigma) for 5 min before the use of the primary antibody. Subsequently, HRP-conjugated secondary antibody (EnVision Flex High pH (Dako, cat# K-80022) was applied for 30 min, followed by the application of 3,3′-Diaminobenzidine (DAB) chromogen (EnVision Flex High pH (Dako, cat# K-80022). The slides were later counterstained with Hematoxylin (cat# 517-28-2, Sigma), and at the end, the slides were mounted with DPX mounting media (cat# 06522, Sigma).

For the analysis of IHC-based staining patterns, semi-quantitative and quantitative approaches were used. The slides were scored by an experienced histopathologist. A semiquantitative expression analysis was carried out for N-cadherin, E-cadherin, vimentin, and androgen receptor expression, where the staining intensity was described as negative (0), weak (+ 1), moderate (+ 2), and strong (3 +) in tumor glands and tumor stromal parts of the PCa tissues. For E-cadherin expression, only tumor glands were analyzed for expression patterns^36–39^. The E-cadherin expression is predominantly cell membranous; however, in tumor cells, sometimes cytoplasmic expression is also seen^40–42^. N-cadherin staining pattern is predominantly cytoplasmic; however, sometimes membranous staining is also seen along with cytoplasmic staining^43^. For vimentin expression, both cytoplasmic and membranous staining patterns were analyzed^39^. Androgen receptor staining primarily involves nuclear staining^44^. Additionally, a staining index was calculated, which involves the multiplication of the staining intensity (0, + 1, + 2, + 3) with the proportion of the positive cells in the PCa tissue slides (0 = 0%, 1 +  = 1–10%, 2 +  = 11–50%, 3 +  =  > 50%^45^. The staining index (SI) is a more sensitive marker of staining intensity patterns since it takes into account both the staining intensity and the proportion of the cells stained. For staining analysis, a light microscope (Olympus BX43, Japan) was used, and the images were captured with a Nikon DS-Fi3 camera (Nikon Corporation, Japan) using Nikon NIS Elements version 5.01 software (Nikon Inc., USA).

For the quantitative analysis of staining intensity and the area stained by specific antibodies (E-cadherin, N-cadherin, Vimentin, and androgen receptor), the IHC profiler plug-in of the ImageJ software was used^46^. This plug-in conducts pixelated analysis of the DAB stained IHC images and reveals the scores into a 4-tier system: negative, low positive, positive, and high positive categories. The IHC profiler assigns pixel intensity values from 0 to 255 based on the predominant pattern of expression. Additionally, the software calculates the mean scores based on the stained pixels in the image.

For statistical analysis, the mean scores of staining intensities, as well as the mean scores of the staining index, were obtained for both EBV-positive and EBV-negative PCa tissues. The Mann–Whitney test was used to determine the statistically significant differences between EBV-positive and EBV-negative PCa tissues (*p* < 0.05).

### Survival benefit analysis in EBV-positive and EBV-negative PCa groups

For the survival benefit analysis, all 99 PCa-positive patients (including both EBV-positive and negative groups) or their next of kin were contacted over the phone. Out of the 99 patients, only 74 patients or their next of kin responded. Therefore, the survival analysis was performed on 74 patients for 38 months, starting from February 2019 (date of sample collection) until February 2022, using the Log-rank (Mantel-Cox) test^47^ and Cox Proportional Hazard Model^48^.

### Institutional review board statement

The study was conducted in accordance with the Declaration of Helsinki and approved by the Ethics Committee of Aga Khan University (AKU-ERC #: 2021-1460-18525).

### Informed consent statement

Informed consent was obtained from all subjects involved in the study.

## Results

### Distribution of perineural invasion in EBV-positive and EBV-negative PCa tissue samples

**Figure 1:**
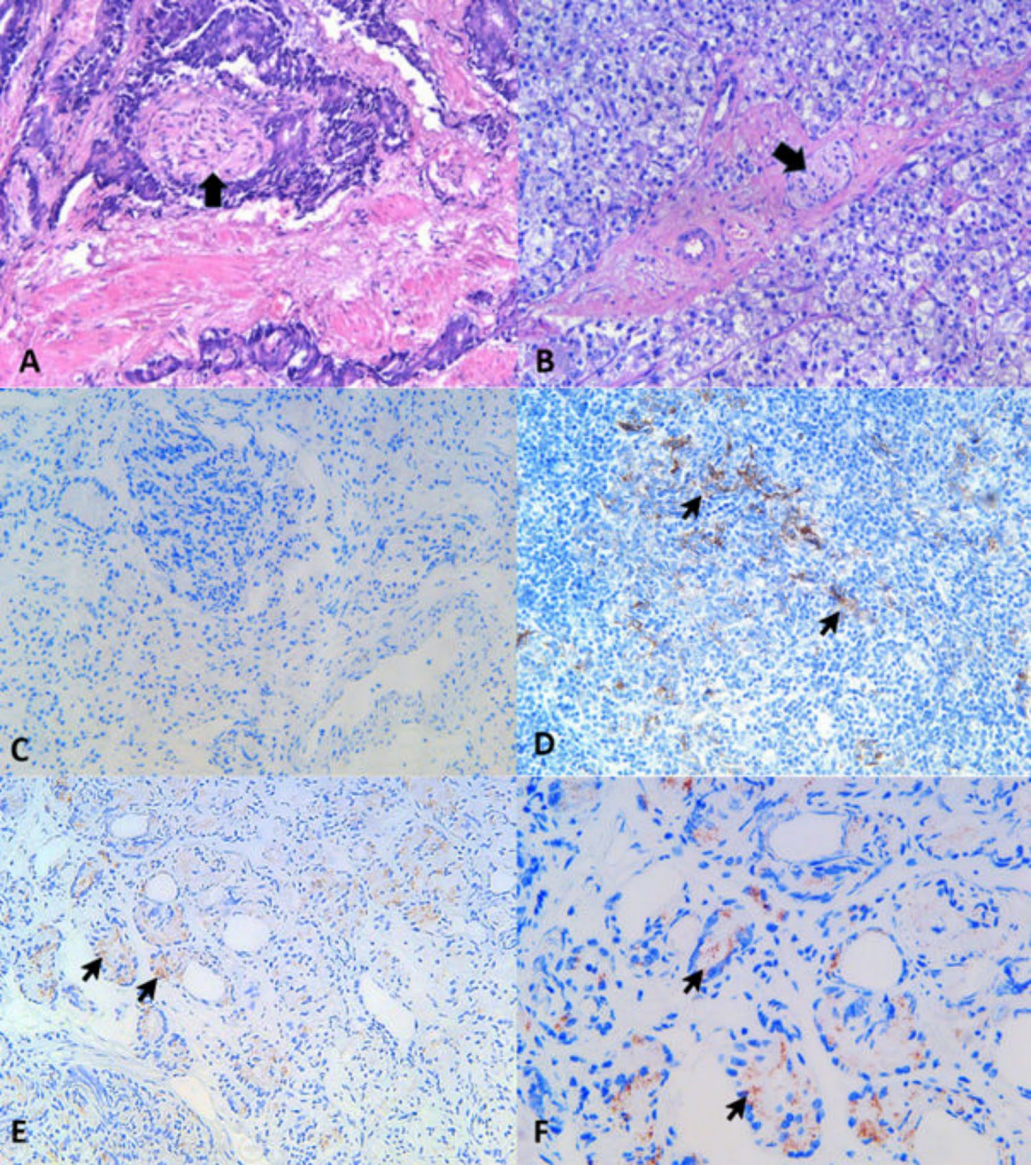
Representative histopathological images showing the presence or absence of perineural invasion in EBV-positive and EBV-negative PCa samples. Representative histopathological images of (**A**) EBV-positive PCa, Gleason score of 9 (4 + 5), showing the crowded cribriform glands infiltrating the perineurium of the nerve in the center, indicating the presence of perineural invasion (PNI) (marked by black arrow). (H&E; original magnification: ×200), and (**B**) EBV-negative PCa, Gleason score of 8 (4 + 4), showing the poorly formed glands with hypernephroid cells. The center shows an un-involved nerve (PNI is absent) marked with an arrowhead (H&E; original magnification: ×200). (**C**–**F**) Immunohistochemical staining of LMP1 in PCa tissues. The figure shows results for LMP1 expression in the (**C**) EBV negative PCa tissue sample, (**D**) Positive control: EBV-associated nasopharyngeal carcinoma sample with confirmed EBV LMP1 expression (black arrows), (**E**, **F**) PCa tissue samples showing weak-moderate EBV LMP1 staining (black arrows) in the tumor cells. The presence of granular cytoplasmic LMP1 immunostaining was labeled as positive. Original magnification ×200 in C, D, E, and ×400 in F.

Descriptive analysis for the distribution of perineural invasion in EBV-positive (n = 39) and EBV-negative (n = 60) PCa samples showed that a higher percentage of EBV-positive PCa (80%) had perineural invasion compared to EBV-negative PCa (67.3%) samples (Fig. 1A, B). This finding was further supported by correlation analysis, which showed a moderate positive correlation between Gleason score (total) and perineural invasion in EBV-positive PCa tissues (r = 0.48; *p* < 0.0147) compared to the EBV-negative group (r = 0.20; *p* > 0.2092) (Table 1).

**Table 1:** Correlation of perineural invasion with Gleason scores in EBV-positive and EBV-negative PCa samples.

Variables	Gleason major scores in EBV-positive samples	Gleason minor scores in EBV-positive samples	Gleason total scores in EBV-positive samples	Perineural invasion in EBV-positive
EBV-positive Gleason major	–	0.4439 (95% CI 0.04689 to 0.7199), *p* < 0.0262*	0.8505 (95% CI 0.6792 to 0.9339), *p* < 0.0001*	0.3325 (95% CI − 0.08433 to 0.6504), *p* < 0.1043
EBV-positive Gleason minor	0.4439 (95% CI 0.04689 to 0.7199), *p* = 0.0262*	–	0.8371 (95 CI 0.6534 to 0.9277), *p* < 0.0001*	0.4599 (95% CI 0.06690 to 0.7294), *p* < 0.0207*
EBV-positive Gleason total	0.8505 (95% CI 0.6792 to 0.9339), *p* < 0.0001*	0.8371 (95 CI 0.6534 to 0.9277), *p* < 0.0001*	–	0.4818 (95% CI 0.09486 to 0.7423), *p* < 0.0147*
Perineural invasion in EBV-positive	0.3325 (95% CI − 0.08433 to 0.6504), *p* < 0.1043	0.4599 (95% CI 0.06690 to 0.7294), *p* < 0.0207*	0.4818 (95% CI 0.09486 to 0.7423), *p* < 0.0147*	–

Variables	Gleason major scores in EBV-negative samples	Gleason minor scores in EBV-negative samples	Gleason total scores in EBV-negative samples	Perineural invasion in EBV-negative
EBV-negative Gleason major	–	0.5148 (95% CI 0.2653 to 0.6998), *p* < 0.0002*	0.7994 (95% CI 0.6637 to 0.8841), *p* < 0.0001*	0.2808 (95% CI − 0.008949 to 0.5271), *p* < 0.0506
EBV-negative Gleason minor	0.5148 (95% CI 0.2653 to 0.6998), *p* < 0.0002*	–	0.9099 (95% CI 0.8423 to 0.9493), *p* < 0.0001*)	0.06070 (95% CI − 0.2324 to 0.3437), *p*- = 0.6786
EBV-negative Gleason total	0.7994 (95% CI 0.6637 to 0.8841), *p* < 0.0001*	0.9099 (95% CI 0.8423 to 0.9493), *p* < 0.0001*	–	0.1826 (95% CI − 0.1124 to 0.4480), *p* < 0.2092
Perineural invasion in EBV-negative	0.2808 (CI − 0.008949 to 0.5271), *p* + 0.0506	0.06070 (95% CI − 0.2324 to 0.3437), *p* = 0.6786	0.1826 (95% CI − 0.1124 to 0.4480), *p* < 0.2092	–

The table displays the correlation coefficients observed between various histopathological parameters (Gleason scores and perineural invasion) in EBV-positive and EBV-negative PCa samples. The r values (Spearman correlation) are presented with 95% confidence interval values, followed by calculated *p*-values. The statistically significant r values (*p* < 0.05) are indicated with*.

### Lymphocytic infiltration status and characterization of T and B cell lymphocytic infiltration in EBV-positive and EBV-negative PCa tissues

**Figure 2:**
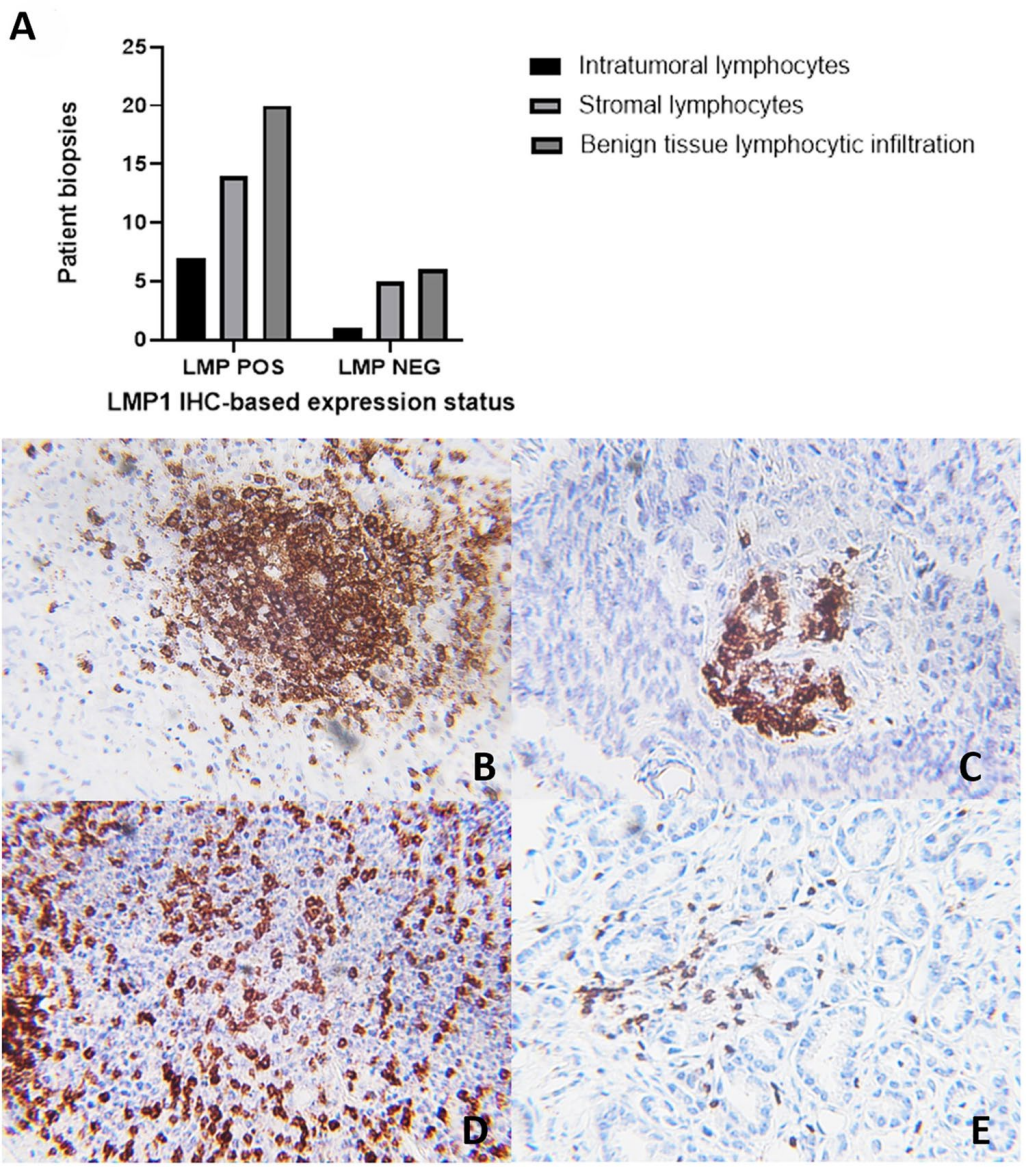
(**A**) Lymphocytic infiltration in EBV LMP1 positive and negative PCa tissues: Y-axis shows the number of PCa tissues, while the x-axis shows the LMP1-positive (POS) and -negative (NEG) groups showing intratumoral, stomal, and adjacent benign tissue lymphocytic infiltration in PCa samples. (**B**–**E**) Representative histopathological images for T and B cell infiltration in EBV-positive and EBV-negative prostate cancer tissues: (**B**) tumor stromal (TS) lymphocytic infiltration of B cells showing formation of lymphocytic aggregations in EBV-negative PCa tissues (3 +) (Original magnification: ×400). (**C**) intratumoral (ITL) B cell lymphocytic infiltration in EBV-positive PCa tissues (2 +) (×400). (**D**) tumor stromal (TS) T cell lymphocytic infiltration in EBV-negative PCa tissues (3 +) (×400) (**E**) intratumoral (ITL) T cell lymphocytic infiltration in EBV-positive PCa tissues (2 +) (×400).

The Hematoxylin and Eosin-based lymphocytic infiltration analysis in PCR EBV-positive versus PCR EBV-negative tissues showed a comparable distribution of lymphocytic infiltration. The comparative descriptive statistical analysis of T and B cell lymphocytic infiltration showed a trend of a higher number of samples with intratumoral and tumor stromal lymphocytic infiltration in EBV-negative tissues compared with EBV-positive tissues (Table 2, Fig. 2B–E).

**Table 2:** Distribution of T and B cell lymphocytic infiltration in intratumoral (ITL) and tumor stroma (ST) in EBV-positive versus EBV-negative PCa tissues.

Descriptive statistics	ITL-0	ITL-1	ITL-2	ITL-3	TS0	TS1	TS2	TS3	ITL (mean with CI)	TS (mean with CI)
A. T lymphocytes (CD3)										
EBV POS	0% (0)	33.33% (12)	41.67% (15)	25% (9)	0% (0)	26.47% (9)	29.41% (10)	44.11% (15)	1.917 (1.656–2.177)	2.176 (1.886–2.467)
EBV NEG	0% (0)	30.77% (12)	41.02% (16)	28.20% (11)	0% (0)	23.68% (9)	28.95% (11)	47.37% (18)	1.974 (1.722–2.226)	2.237 (1.967–2.506)
B. B lymphocytes (CD20)										
EBV POS	32.35% (11)	35.29% (12)	8.82% (3)	23.53% (8)	20.59% (7)	20.59% (7)	5.88% (2)	52.94% (18)	1.235 (0.8319–1.639)	1.912 (1.471–2.353)
EBV NEG	30.55% (11)	36.11% (13)	8.33% (3)	25% (9)	18.92% (7)	21.62% (8)	5.40% (2)	54.05% (20)	1.278 (0.8848–1.671)	1.946 (1.531–2.361)

Tables (A) CD3 (B) CD20 show the frequency and distribution of ITL and ST lymphocytic infiltrations in EBV-positive and EBV-negative PCa tissues.

In the next step, logistic regression analysis was performed to examine the influence of EBV status on different variables. The results showed that the model as a whole was significant (Chi2(1) = 8.55, *p* = 0.003). The regression analysis showed that among all analyzed variables, only CD3 intratumoral lymphocytic infiltration was found to be significantly associated with EBV-positive status. EBV-positive status was associated with decreased odds of having CD3 intratumoral lymphocytic infiltration in PCa tissues (OR = 0.07; 95% CI 0.01–0.65, *p*-value < 0.019).

We also performed a subset analysis on IHC-based LMP1 expression-positive (n = 22) and LMP1 expression-negative (n = 9) EBV PCR-positive tissues, which showed higher lymphocyte infiltration in LMP1 expression-positive compared to LMP1 expression-negative tissues (Figs. 2A).

### Differential mRNA expression analysis of selected oncomiRs and genes associated with oncogenesis and EMT in EBV-positive and EBV-negative PCa tissues.

**Figure 3:**
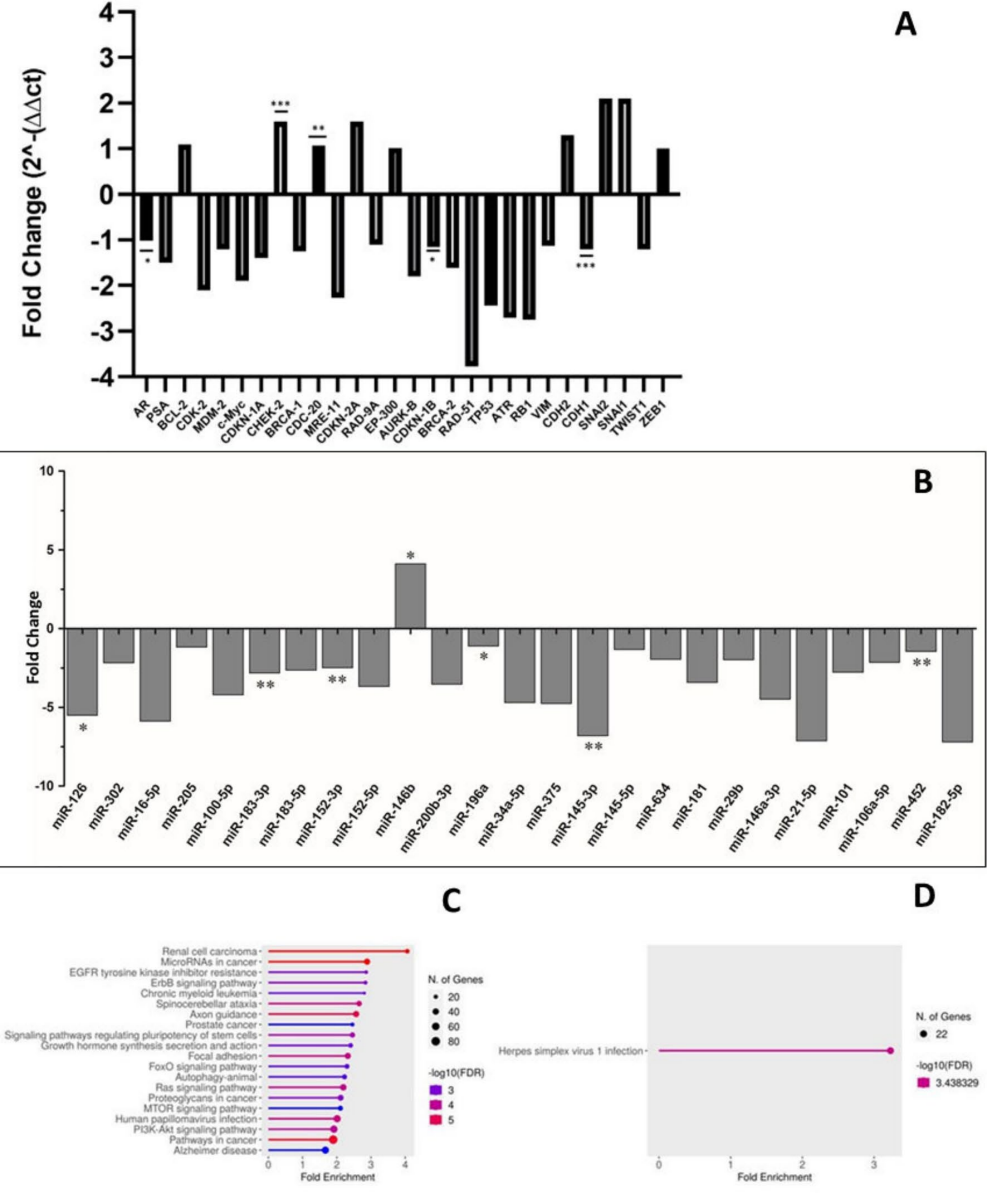
Differential expression of oncogenes and EMT-associated genes in EBV-positive and EBV-negative PCa samples: (**A**) Fold change of selected oncogenes (*c-MYC, AURKB, CDC20, EP300, MDM2, TP53, RB1, CHEK2, CDKN-1B, CDKN2A, BRCA1, BRCA2, RAD51, CDK2, CDKN1A, RAD9A, MRE11, ATR, BCL2, KLK3,* and *AR*) and EMT-associated markers (*CDH1, CDH2, VIM, SNAI2, SNAI1, TWIST1, and ZEB1*) in EBV-positive and EBV-negative PCa samples. Fold changes of the genes exhibiting statistical significance are annotated with asterisk (*p* < 0.05). (**B**) Fold change expression of twenty-five differentially expressed prostate-specific miRNAs in EBV-positive and -negative PCa tissues. Fold change expression of 25 prostate-specific miRNAs in EBV-positive and -negative PCa tissues. The miRNAs exhibiting statistically significant (*p* < *0.05*) differences in their expression between EBV-positive and -negative PCa tissues are marked with an asterisk. (**C**, **D**) Pathway enrichment analysis gene targets for differentially expressed miRNAs in EBV-positive and EBV-negative PCa tissues.

The differential mRNA expression of EMT-associated transcripts showed a statistically significant difference only in the expression of *CDH1* (E-cadherin; *p*-value < 0.0004) in EBV-positive and EBV-negative tissues (Fig. 3A ; Figure S1), where the expression of *CDH1* was found to be 1.2-fold lower in EBV-positive PCa tissues compared to EBV-negative PCa tissues (Fig. 3A; Table S1). The expression of *VIM* (Vimentin) was 1.12-fold lower in EBV-positive PCa tissues when compared with EBV-negative PCa tissues, although the difference in expression between the EBV-positive and EBV-negative groups was not statistically significant.

Differential mRNA expression of selected genes involved in PCa oncogenesis (*c-MYC, AURKB, CDC20, EP300, MDM2, TP53, RB1, CHEK2, CDKN-1B, CDKN2A, BRCA1, BRCA2, RAD51, CDK2, CDKN1A, RAD9A, MRE11, ATR, BCL2, PSA,* and *AR*) showed statistically significant differences only in the expression of *AR* (EBV-positive: 2.02, EBV-negative: 1.99; *p* = 0.01), *CHEK-2* (EBV-positive: − 0.35, EBV-negative: 0.36; *p* = 0.001), *CDKN-1B* (EBV-positive: 1.90, EBV-negative: 1.69; *p* = 0.03), and *CDC-20* (EBV-positive: − 0.02, EBV-negative: 0.08; *p*-value = 0.003) (Fig. 3A; Table S1). The expression of *AR, CHEK-2, CDKN-1B*, and *CDC-20* was found to be, respectively, 1.02-fold lower, 1.6-fold higher, 1.15-fold lower, and 1.07-fold higher in EBV-positive PCa tissues compared to EBV-negative PCA tissues.

Similarly, the differential miRNA expression analysis of prostate-specific oncomiRs showed statistically significant differences only in the expression of *miR-126, miR-152-3p, miR-452, miR-145-3p, miR-196a, miR-183-3p,* and *miR-146b* that exhibited, respectively, 5.5-fold lower, 2.4-fold lower, 1.4-fold lower, 6.8-fold lower, 1.1-fold lower, 2.8-fold lower, and 4.11-fold higher expression in EBV-positive PCa tissues compared to EBV-negative PCa tissues (Fig. 3B).

### Pathway enrichment analysis of gene targets for differentially expressed miRNAs in EBV-positive and EBV-negative PCa tissues

In the next step, we examined the pathway enrichment for gene targets of upregulated and down-regulated miRNAs within EBV-positive tissues. For upregulated miRNAs in EBV-positive PCa tissues, we observed enrichment of pathways related to renal cell carcinoma (RCC), miRNAs in cancer, prostate cancer, chronic myeloid leukemia, Ras, MTOR, and PI3K-Akt signaling pathways, among others (Fig. 3C). For downregulated miRNAs in EBV-positive PCa tissues, enrichment was observed only in Herpes simplex virus I (HSV-1) infection (Fig. 3D).

### Immunohistochemistry-based expression analysis of AR and EMT markers in EBV-positive and EBV-negative PCa tissues

**Figure 4:**
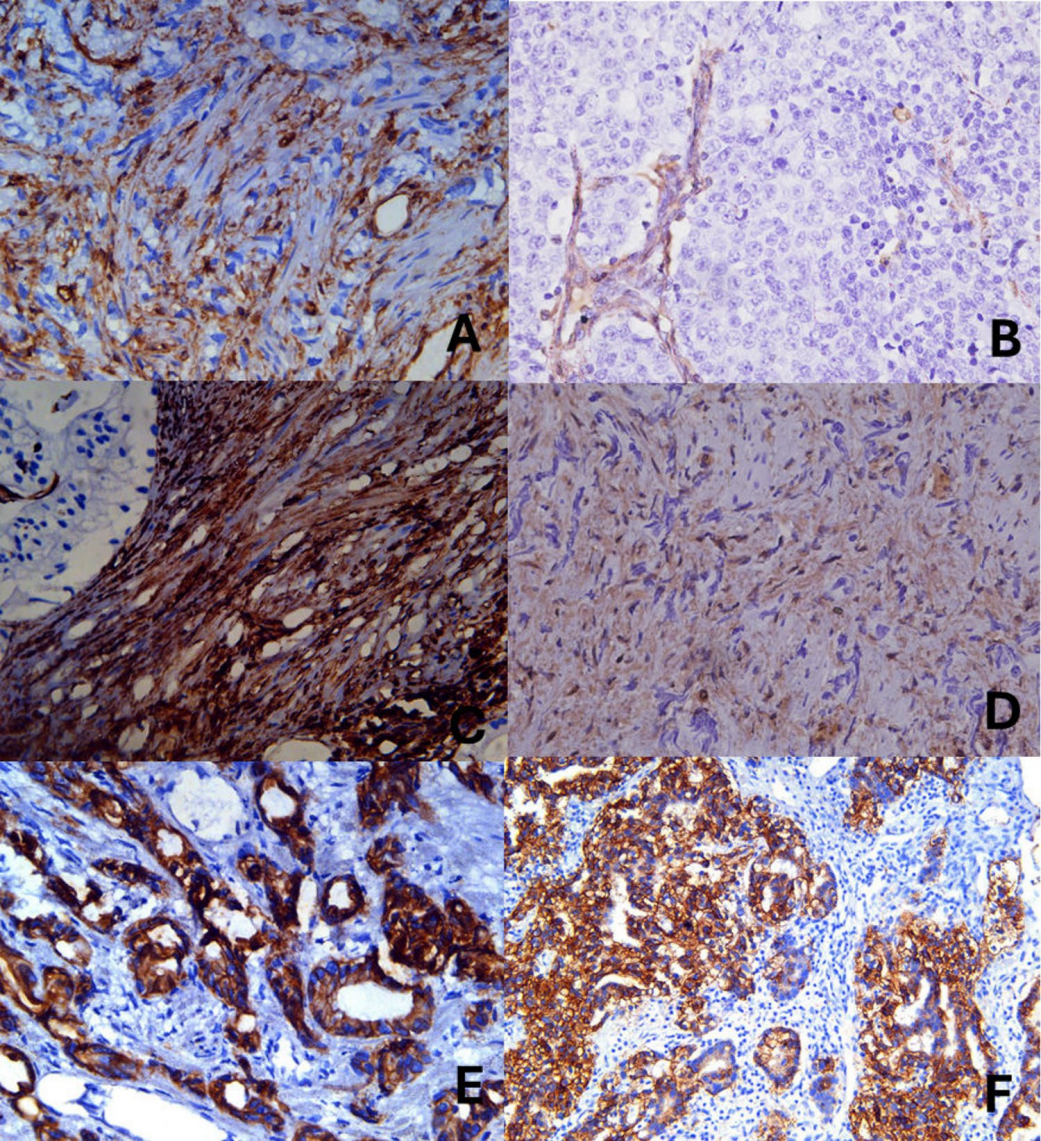
Representative histopathological images of IHC-based vimentin protein expression: (**A**) Tumor gland with moderate (+ 2) cytoplasmic staining intensity pattern in EBV-positive PCa tissue (Original magnification ×400). (**B**) Tumor gland with vimentin-negative expression in EBV-negative PCa tissues. ×400. (**C**) EBV-positive PCa tumor stroma with strong (+ 3) cytoplasmic staining intensity pattern, ×400. (**D**) EBV-negative PCa tumor stroma with moderate (+ 2) staining intensity, ×400. (**E**, **F**) Representative histopathological images of IHC-based E-Cadherin protein expression; (**E**) Tumor gland with strong (+ 3) cytoplasmic and membranous intensity pattern in EBV-positive PCa tissue (Original magnification ×400), and (**F**) EBV-negative tumor gland with moderate (+ 2) staining intensity pattern in EBV-negative PCa tissue, tumor stroma is negative for E-cadherin expression on right half of the image, ×200.

The descriptive statistical analysis of IHC-based protein expression of vimentin in both tumor glandular and tumor stromal parts of tissues revealed stronger intensity patterns in EBV-positive tissues (n = 39) compared to EBV-negative (n = 60) PCa tissues. For instance, 30% of EBV-positive PCa tissue had moderate (+ 2) to strong (+ 3) staining intensity in tumor glands, compared to 12% of EBV-negative PCa samples (Fig. 4A–D; Table S5). Similarly, 68% of EBV-positive tissues exhibited a strong (+ 3) vimentin staining intensity pattern in tumor stroma, compared to EBV-negative tissues.

**Figure 5:**
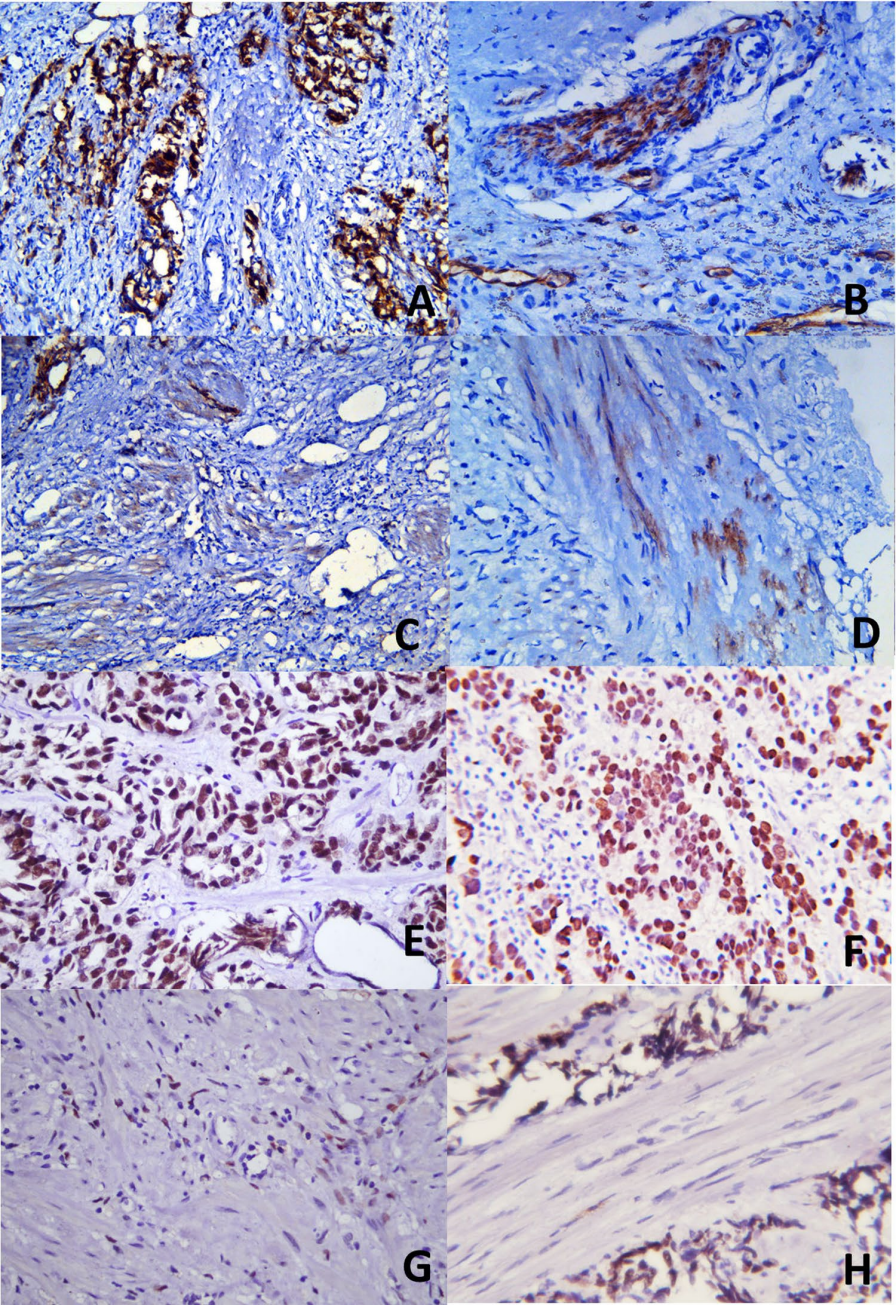
Representative histopathological images of IHC-based N-Cadherin protein expression. (**A**) Tumor gland with strong (+ 3) cytoplasmic and membranous staining intensity pattern in EBV-positive PCa tissue. (Original magnification ×200). (**B**) Tumor gland with weak (+ 1) staining intensity expression with cytoplasmic granular pattern in EBV-negative PCa tissues. ×400. (**C**) EBV-positive PCa with weak (+ 1) staining intensity with cytoplasmic pattern. ×200. (**D**) EBV-negative PCa tumor stroma with weak (+ 1) staining intensity with cytoplasmic granular pattern of expression. ×400. (**E**–**H**) Representative histopathological images of IHC-based androgen receptor protein expression, (**E**) Tumor gland with strong (+ 3) nuclear staining intensity pattern in EBV-positive PCa tissue. (Original magnification ×400). (**F**) Tumor gland with moderate (+ 2) nuclear staining intensity pattern in EBV-negative PCa tissues. ×400. (**G**) EBV-positive PCa tumor stroma with negative androgen receptor expression (lower half of the image). ×400. (**H**) EBV-negative PCa tumor stroma with moderate (+ 2) staining intensity. ×400.

For E-cadherin and N-cadherin, the staining intensity patterns were comparable in EBV-positive and EBV-negative PCa tissues (Figs. 4E, F and 5A–D; Table S5). Similarly, the E-Cadherin to N-Cadherin ratio was also found to be comparable in both groups (EBV-Positive: 2.286/1.286 = 1.78; EBV-negative: 1.871/1.152 = 1.62). These findings were further supported by the ImageJ-based analysis, which showed that EBV-positive PCa tissues had an overall higher expression (75%) of vimentin compared to EBV-negative PCa tissues (42%).

For androgen receptor expression, the results showed a higher percentage (23.08%) of EBV-positive PCa tissues had weak (+ 1) staining intensity compared to EBV-negative (12.90%) PCa tissues (Fig. 5E–H; TableS5). The staining intensity patterns in the moderate to strong categories were comparable in both groups. The staining intensity patterns in tumor stroma showed that a higher percentage of EBV-positive samples showed AR-negative expression (83.33%) compared to EBV-negative samples (76%).

The statistical analysis based on the scores of the staining index and IHC profiler-based quantified assessment of staining intensity patterns in Vimentin, E-cadherin, N-cadherin, and androgen receptors showed that there were statistically significant differences only in the expression of vimentin in tumor stroma and E-cadherin expression in the tumor glands between EBV-positive and EBV-negative tissues (Table 3; Table S4).

**Table 3:** IHC profiler-based quantified assessment of staining intensity patterns in Vimentin, E-cadherin, N-cadherin, and androgen receptor.

	EBV-positive PCa tissues (mean with SD)	EBV-negative PCa tissues (mean with SD)	Statistically significant difference *p*-value (*p* < 0.05) with 95%CI (confidence interval) Mann Whitney test
Vimentin			
PCa glands (Staining intensity)	0.7692 ± 1.210	0.3030 ± 0.8472	0.0912 (0.000 to 0.000)
	N = 26	N = 33	
PCa glands (Staining index)	4.000 ± 3.117	4.250 ± 3.202	0.6566 (− 6.000 to 6.000)
	N = 8	N = 4	
PCa tumor stroma (Staining intensity)	2.320 ± 1.145	1.625 ± 1.185	0.0067* (− 1.000 to 0.000)
	N = 25	N = 32	
PCa tumor stroma (Staining index)	6.900 ± 1.971	3.364 ± 1.620	< 0.0001* (− 4.000 to 3.000)
	N = 20	N = 22	
E-cadherin			
PCa glands (Staining intensity)	2.286 ± 0.6587	1.871 ± 0.4995	0.0052* (− 1.000 to 0.000)
	N = 28	N = 31	
PCa glands (Staining index)	6.880 ± 2.261	5.600 ± 1.522	0.0063* (− 3.000 to 0.000)
	N = 25	N = 30	
N-cadherin			
PCa glands (Staining intensity)	1.286 ± 1.013	1.152 ± 0.9722	0.6445 (− 1.000 to 0.000)
	N = 28	N = 33	
PCa glands (Staining index)	2.700 ± 2.227	2.909 ± 2.328	0.6045 (0.000 to 1.000)
	N = 20	N = 22	
PCa tumor stroma (Staining intensity)	1.071 ± 0.8997	0.9091 ± 0.8048	0.5165 (− 1.000 to 0.000)
	N = 28	N = 33	
PCa tumor stroma (Staining index)	1.900 ± 0.8522	1.909 ± 1.192	0.6647 (− 1.000 to 0.000)
	N = 20	N = 22	
Androgen receptor			
PCa glands (Staining intensity)	2.000 ± 0.6928	2.097 ± 0.5975	0.6687 (0.000 to 0.000)
	N = 26	N = 31	
PCa glands (Staining index)	5.654 ± 2.399	6.194 ± 1.905	0.4426 (0.000 to 0.000)
	N = 26	N = 31	
PCa tumor stroma (Staining intensity)	0.2083 ± 0.5090	0.3200 ± 0.6272	0.6420 (0.000 to 0.000)
	N = 24	N = 25	
PCa tumor stroma (staining index)	1.750 ± 0.9574	2.000 ± 1.915	0.9999 (− 2.000 to 3.000)
	N = 4	N = 7	

It shows the statistical analysis of the quantified scores of staining intensity and proportion of stained cells of vimentin, E-cadherin, N-cadherin, and androgen receptor in tumor glands and tumor stroma of EBV-positive and EBV-negative PCa tissues. Only the statistically significant values (*p* < 0.05) are indicated with*.

### Survival analysis of EBV-positive and EBV-negative prostate cancer patient groups

For the EBV-positive and EBV-negative prostate carcinoma groups, the percentage of survival over 38 months was calculated using the log-rank test and Cox Proportional Hazard Model. The statistical analysis shows that the presence of EBV does not confer any survival disadvantage to the patient, and the survival was comparable in patients based on the EBV status of their PCa tissue samples (Table 4; Figure S2).

**Table 4:** Survival analysis of EBV-positive and EBV-negative prostate cancer patient groups.

	Chi-square	df	*p*						
(A) Log-rank									
Log rank	0.05	1	0.818						
(B) Cox proportional hazard model									
Overall	0.06	1	0.808						

Name	Coefficients	Lower 95% CI	Upper 95% CI	Std. error	z	*p*	Exp(B)	Lower 95% CI	Upper 95% CI
Status EBV POS	− 0.12	− 1.07	0.83	0.49	0.24	.809	0.89	0.34	2.3

It shows the results from log-rank analysis (A) and Cox proportional hazard model (B).

## Discussion

In this study, two key histopathological markers of PCa aggressiveness, namely perineural invasion (PNI) and lymphocytic infiltration, were assessed^49,50^. The analysis of perineural invasion in EBV-positive and EBV-negative PCa samples showed a higher percentage of EBV-positive PCa (80%) had PNI compared to EBV-negative PCa samples (67.3%), and a statistically significant association was found between positive EBV status and the presence of PNI (Table 1), further strengthening the possibility that EBV infection might be associated with aggressive forms of PCa. These findings are clinically important as high Gleason scores and PNI are associated with an aggressive form of PCa^51–54^. Studies have reported that EBV-positive PCa samples tend to have higher Gleason scores compared to EBV-negative PCa tissues^4^. The aggressive nature of PCa in patients with high Gleason scores is associated with the expression of EMT-associated genes^55,56^, which histopathologically manifests as the presence of perineural invasion in PCa samples^50,57^. EBV has frequently been associated with epithelial cancers in humans, such as EBV-associated gastric carcinomas and EBV-associated nasopharyngeal carcinomas of the non-keratinizing subtype^58,59^. Furthermore, EBV is known to promote metastasis in nasopharyngeal carcinoma via EMT^10^, however, nothing is known about the role of EBV in PCa and its association with perineural invasion and EMT. Studies have reported that EBV-positive nasopharyngeal carcinoma cases tend to be more invasive, with early metastasis, compared to EBV-negative nasopharyngeal carcinoma cases, and these features are mediated by EBV latent membrane protein-1 (LMP1)^60^. At the same time, it has been reported that EBV-positive nasopharyngeal carcinoma cases are more responsive to radiotherapy and chemotherapy compared to EBV-negative NPC cases^61^.

This study also shows the presence of higher lymphocytic infiltration in LMP1-positive prostate cancer tissues (Fig. 2). It has been reported that EBV-positive nasopharyngeal carcinoma samples had a significantly higher number of tumor-infiltrating lymphocytes compared with EBV-negative nasopharyngeal carcinoma samples^62^, and the presence of a higher number of tumor-infiltrating lymphocytes is associated with better survival in nasopharyngeal carcinoma patients^63^. Similarly, better survival is associated with the presence of tumor-infiltrating lymphocytes in EBV-associated gastric carcinoma^64^. Tumor-infiltrating lymphocytes have an important role in prostate carcinoma^65^. The characterization of T and B lymphocytes showed that a higher number of EBV-negative PCa tissues had lymphocytic infiltration compared to EBV-positive PCa tissues, which specifically exhibited a lower number of CD3 ITL (Table 2). Our findings are further supported by other studies showing that the presence of EBV in cancer tissues leads to the suppression of T-cell immune responses via EBV-encoded proteins^66,67^. However, this phenomenon has not been explored in detail in the PCa, and further investigations are required to explore how EBV might lead to intratumoral lymphocytic infiltration suppression.

In the next step, we determined the IHC-based expression of EMT markers, namely E-cadherin, N-cadherin, and Vimentin, associated with the progression of PCa^68^, along with AR expression in EBV-positive and -negative PCa tissues (Table S5). We found statistically significant differences in the protein expression intensity patterns of Vimentin and E-cadherin in EBV-positive and EBV-negative PCa tissues (Table 3). It has been reported that high IHC-based expression of Vimentin is associated with the enhanced invasiveness and distant metastasis of PCa tumor cells, including bone metastasis^69,70^. Furthermore, in prostate cancer cell lines, the enhanced expression of Vimentin promoted tumor cell invasiveness^71^, which showed a positive correlation with invasion and metastasis in the context of androgen-independent PCa^39^. At the mechanistic level, the expression of EBV latency genes, i.e., EBNA1 or EBNA3C, is associated with the upregulation of Vimentin and its subsequent association with tumor metastasis involving EMT^72^. In nasopharyngeal carcinoma, early tumor metastasis is linked with the EBV LMP1-mediated increase in the expression of Vimentin^73^, and high Vimentin expression is often significantly associated with the advanced clinical stage and lymph node metastasis in nasopharyngeal carcinoma tissues^74^.

Aberrant expression of E-cadherin, another marker of EMT-associated tumor progression, is associated with the progression of PCa and metastasis of the disease^42^. In our study, the statistical analysis of the mean values of staining intensity of E-cadherin showed statistically significant differences in EBV-positive PCa tissues (2.286) compared to EBV-negative PCa tissues (1.871). It is reported that reduced expression of E-cadherin is often associated with the progression of PCa and poor prognosis in patients^45,75^. However, in our study, we found higher mean scores of E-cadherin intensities in EBV-positive PCa tissues as compared to EBV-negative PCa tissues. In the case of EBV-associated gastric cancer, no differences were found in the expression of E-cadherin in EBV-positive versus EBV-negative gastric cancer^76^. In contrast, it is documented that in the case of EBV-associated nasopharyngeal carcinoma, the expression of E-cadherin is reduced and is a marker of EMT^77^.

The gene expression analysis showed that the expression of N-cadherin (*CDH2*) was 1.3-fold higher (*p* > 0.05), and E-cadherin (*CDH1*) was 1.2-fold lower (*p* < 0.05) in EBV-positive PCa tissues compared to EBV-negative PCa tissues, respectively (Fig. 3A; Table S1). The upregulation of N-cadherin and downregulation of E-cadherin are hallmarks of EMT^78^ and may indicate that EBV may have a role in the progression of prostate carcinoma through the expression of EMT-associated hallmark genes. EMT plays a critical role in tumorigenesis and strong association with increased tumor invasion and metastasis^79^. These findings are in agreement with the reported literature, where upregulation of N-cadherin has shown to be associated with metastasis and poor prognosis of PCa^80^. Although nothing is known about the role of EBV LMP1 in PCa, it has been shown to play a crucial role in mediating EMT and enhancing metastasis and invasion in nasopharyngeal carcinoma^11,81^. However, we did not find statistically significant differences in the gene expression of vimentin in EBV-positive versus EBV-negative PCa tissues. Further pathway analysis is required to further elucidate the underlying mechanisms involving the role of EBV and its association with EMT in PCa patients.

In addition to EMT genes, the expression of four other genes, namely *AR*,* CHEK-2*, *CDKN-1B*, and *CDC-20*, implicated in the oncogenesis of PCa, was found to be significantly different (*p* < 0.05) in EBV-positive compared to EBV-negative PCa tissues (Fig. 3A; Table S1). These findings are important because of the critical roles these genes play in PCa carcinogenesis^26,82^. The androgen receptor plays a critical role in the carcinogenesis of prostate carcinoma as well as in the acquisition of the castration-resistant PCa phenotype^83^. We found a 1.02-fold less AR gene expression in EBV-positive PCa tissues compared with EBV-negative tissues. These findings are consistent with reported studies where the dysregulation of AR, specifically decreased expression of the androgen receptor, results in the acquisition of metastatic castration-resistant PCa phenotype associated with the progression of PCa^83,84^. Furthermore, HHV-8, another oncogenic herpesvirus, has been shown to derive the acquisition of an androgen-insensitive phenotype in prostate cell lines with altered AR expression via EZH2-mediated silencing of *DAB2IP* and *MSMB*^85^. However, the mechanism for the EBV-derived downregulation of AR gene expression in PCa is unknown.

Uncontrolled cell proliferation as a result of the dysregulation of the cell cycle is often associated with the onset/progression of cancer, resulting in neoplastic changes in the cells^86^. EBV latency involves the expression of EBV latency-associated genes^4^, which often result in the dysregulation of the cell cycle, causing unregulated progression of the G1/S phase and inhibition of apoptosis^87^. Checkpoint kinase 2 (*CHEK-2*) is a tumor suppressor gene that encodes for serine-threonine kinase (CHK2) and is involved in the regulation of apoptosis, cell cycle arrest, and DNA repair^88^. We found a 1.6-fold higher expression of *CHEK-2* in EBV-positive PCa tissues compared to EBV-negative PCa samples, indicating that the presence of EBV in the tissue might be associated with the dysregulation of *CHEK-2* gene expression. *CDKN-1B* is a tumor suppressor gene that encodes for the inhibitor of the cell cycle p27^Kip1^, which is a cell cycle inhibitor protein^89^. Studies have shown that* CDKN-1B* is a tumor suppressor gene and its dysregulated gene expression, leading to the loss of cell cycle control, is associated with the progression of PCa^90,91^. We found a 1.15-fold lower expression of *CDKN-1B* in EBV-positive PCa tissues compared to EBV-negative PCa samples. Studies have reported that the loss of *CDKN-1B* expression is associated with the progression of PCa in tumors with low Gleason scores^92,93^. Similarly, the *CDC-20* gene encodes for the cell division cycle protein 20 homolog, which regulates the cell cycle by activating the anaphase-promoting complex (APC/C)^94^. We found a 1.07-fold higher expression of *CDC-20* in EBV-positive PCa tissues compared to EBV-negative PCa tissues. Studies have reported that the overexpression of *CDC-20* is associated with aggressive early-onset metastatic PCa with a poor prognosis^95,96^. Moreover, the overexpression of *CDC-20* is associated with PCa treatment failure and the acquisition of treatment-resistant phenotypes^97^. Further studies may be required to better understand* CDC-20* as a potential therapeutic target for the treatment of PCa. In this study, the expressions of other critical oncogenes were comparable between EBV-positive and EBV-negative tissues. It is important to mention here that the comparison was between two groups of cancer tissues that only differed by EBV status. Hence, not observing significant differences in the gene expression profile in both groups is not surprising, as PCa, even in the absence of any viral etiology, does exhibit dysregulation of the expression of important oncogenes such as *Rb* and *TP53*, which are part of PCa oncogenesis^98^. However, further studies need to be conducted to better understand the mechanistic role of prostate carcinoma-associated genes in EBV-positive prostate cancers.

Furthermore, in this study, the differential expression of miRNAs within the PCa tissues showed a statistically significant down-regulation of tumor-suppressive microRNAs, namely *miR-126*, *miR-152-3p*, *miR-452*, *miR-145-3p*, *miR-196a* and *miR-183-3p* in EBV-positive PCa as compared to EBV-negative PCa tissues (Fig. 3B). Our findings suggest that EBV, especially through its LMP-1 proteins, leads to the modulation of miRNA (mostly oncomiRs) that play a significant role in prostate oncogenesis. For instance, a reduced expression of *miR-126,* also observed in EBV-positive PCa tissues in our study, has been associated with the enhanced tumor Epithelial to Mesenchymal Transition (EMT) and metastasis though regulation of Disintegrin and metalloproteinase domain-containing protein 9 (ADAM 9)^99,100^. Similarly, we also found the expression of tumor suppressive *miR-152-3p* to be reduced in EBV-positive PCa tissues. *miR-152-3p* acts as a tumor suppressor along with *miR-148-3p* by synergistically repressing Kruppel-like factor 4 (KLF-4), thereby regulating cell proliferation, differentiation, and migration^101,102^. In addition to this, tumor suppressive *miR-452* also showed reduced expression in EBV-positive PCa tissues. As reported in earlier studies, WW domain-containing E3 ubiquitin protein ligase-1 (WWP-1), a potential driver of oncogenesis and metastasis, is a direct target of *miR-452.* Hence reduced expression of *miR-452* can lead to increased WWP-1 expression and thereby increased PCa cell migration and invasion^102,103^. *miR-145-3p* is another tumor suppressor, exhibiting decreased expression in our EBV-positive PCa tissues, known to regulate PCa cell proliferation, metastasis, and apoptosis via directly targeting Metadherin (MTDH)^104^. MTDH promotes invasion and metastasis through the activation of nuclear factor-κB (NF-κB), interleukin-8 (IL-8), and matrix metalloproteinase-9 (MMP-9)^105^. High-Mobility Group Nucleosome Binding Domain-5 (HMGN-5) is a Histone-1 binding protein, well documented for its role in PCa cell proliferation and metastasis by activation of MAPK pathways and causing resistance to gemcitabine. Tumor suppressive *miR-183-3p* directly regulates the expression of HMGN-5 thereby regulating PCa tumorigenesis and development^106,107^. *miR-146b* has been studied in PCa development due to its dual role as oncomiR and tumor suppresser^108,109^. Increased expression of *miR-146b* inhibits autophagy via the mTOR/AKT signaling pathway, thereby promoting PCa proliferation. In this study, we reported an increased expression of *miR-146b* in EBV-positive PCa tissues. Besides this we also found other tumor suppressive miRNAs downregulated in EBV-positive PCa tissues, yet their expression among EBV-positive and -negative PCa tissues was not statistically significant.

Our analysis of gene ontology and pathway enrichment, conducted on gene targets affected by differentially expressed miRNAs in both EBV-positive and -negative prostate cancer (PCa) tissues, revealed a notable enrichment of genes associated with the development of various cancers and pathogenic infections (Fig. 3C, D). For instance, when examining the pathways enriched in EBV-positive and -negative PCa tissues, we observed that the pathway leading to the control of herpes virus infection was enriched, and the studied miRNAs were downregulated, suggesting poor control of herpes infection and enhanced progression of prostate cancers. This finding is particularly significant given previous research demonstrating a connection between herpesviruses and the development of PCa^110,111^.

Finally, we performed a survival benefit analysis in EBV-positive and EBV-negative PCa patients and found that the EBV status was independent of survival proportions (Table 4; Fig. S2). The role of EBV in the progression of prostate carcinoma has not been previously reported. Previous studies have suggested a conflicting role of EBV in various EBV-associated cancers^112^, such as Song et al.^113^ showed that the presence of EBV conferred a survival advantage to patients with EBV-associated gastric carcinoma, whereas in EBV-associated nonkeratinizing subtype of nasopharyngeal carcinoma, EBV infection has been associated with an increased risk of distant metastasis^114,115^. However, as discussed earlier, the possible prognostic value of EBV-associated lymphocyte infiltration of the prostate tissue may be further investigated for its possible association with the overall survival of patients with EBV-associated PCa as has been reported in cases of EBV-associated nasopharyngeal and gastric carcinoma^63,64^.

There are certain limitations in this study: for the survival analysis, other clinical parameters such as treatment regimens during the period of this analysis could not be included, as this information was not available when these samples were sent for biopsies. Also, there was an imbalance in the number of retrospectively collected EBV-positive and EBV-negative PCa samples, which may introduce some sampling bias^116^. However, this bias may be limited as we considered events (deaths) and not the actual sample number in the analysis. Furthermore, the survival analysis was limited to 38 months, during which time the patients were followed up for this study. Analysis beyond 38 months may provide additional information on survival benefits for the patients. Additionally, due to limited resources, only 20 EBV-positive and 20 EBV-negative PCa samples were included for gene expression analysis. However, the statistical sample size calculations showed that this sample size is sufficiently powered (80%) to explain the differences between the two groups. Given that a limited set of genes (n = 28) were analyzed for differential expression in EBV-positive and EBV-negative PCa tissues, there is a possibility that additional uninvestigated genes involved in PCa oncogenesis may also be differentially expressed in the two groups.

## Conclusion

In conclusion, the presence of EBV may be associated with the progression of prostate carcinoma. Further studies with a larger sample size and mechanistic correlations are required to strengthen the link between EBV, PCa prognosis, and its association with overall patient survival.

### Supplementary Information

Supplementary Information.

## Data Availability

All data is available in the manuscript and its supplementary files.
