# Supplementary material for: Differential analysis of histopathological and genetic markers of cancer aggressiveness, and survival difference in EBV-positive and EBV-negative prostate carcinoma
Source: Sci Rep. 2024 May 5;14:10315. doi: 10.1038/s41598-024-60538-0 (PMC11070424; doi:10.1038/s41598-024-60538-0)
Supplement: Supplementary file 1 — Supplementary Information. [file 41598_2024_60538_MOESM1_ESM.docx]

**Table S1: Forward and reverse primers of prostate cancer-associated genes.**

| **Prostate carcinogenesis-associated genes** | **Primer Sequences (5’-3’)** |
| --- | --- |
| *N-Cadherin* | Fwd: TTTGATGGAGGTCTCCTAACACC  Rev: ACGTTTAACACGTTGGAAATGTG |
| *E-Cadherin* | Fwd: ATTTTTCCCTCGACACCCGAT  Rev: TCCCAGGCGTAGACCAAGA |
| *Vimentin* | Fwd: AGTCCACTGAGTACCGGAGAC  Rev: CATTTCACGCATCTGGCGTTC |
| *Slug* | Fwd: CGAACTGGACACACATACAGTG  Rev: CTGAGGATCTCTGGTTGTGGT |
| *Snail* | Fwd: TCGGAAGCCTAACTACAGCGA  Rev: AGATGAGCATTGGCAGCGAG |
| *Twist* | Fwd: GTCCGCAGTCTTACGAGGAG  Rev: GCTTGAGGGTCTGAATCTTGCT |
| *Zeb 1/2* | Fwd: TTACACCTTTGCATACAGAACCC  Rev: TTTACGATTACACCCAGACTGC |
| *Β-Actin* | Fwd: GTCTGCCTTGGTAGTGGATAATG  Rev: TCGAGGACGCCCTATCATGG |
| *AURKB* | Fwd: CAGAAGAGCTGCACATTTGACG  Rev: CCTTGAGCCCTAAGAGCAGATTT |
| *CDC-20* | Fwd: CAGAAGAGCTGCACATTTGACG  Rev: CCTTGAGCCCTAAGAGCAGATTT |
| *EP300* | Fwd: TTCCCCTAACCTCAATATGGGAG  Rev: GCCTGTGTCATTGGGCTTTTG |
| *MDM2* | Fwd: TCGTCGGGTGAGGGTACTG  Rev: AACCACTTCTTGGAACCAGGT |
| *TP53* | Fwd: AGCTTGATCGCCTCTATAAGGA  Rev: CCCTCAGCTCATTAACACGCT |
| *RB-1* | Fwd: GACCCAGAAGCCATTGAAATCT  Rev: GGTGTGCTGGAAAAGGGTCC |
| *CHEK-2* | Fwd: TGAGAACCTTATGTGGAACCCC  Rev: ACAGCACGGTTATACCCAGC |
| *CDKN-1B* | Fwd: TCTTCATTCACACCGAGTAGTGC  Rev: TGAGGTTAGAGCCATCTGGAAA |
| *CDKN2A* | Fwd: GTGTATAGGGTCGGCCATCAA  Rev: CCTGCCGTTGTTACCTGAGAG |
| *BRCA-1* | Fwd: GCTCGTGGAAGATTTCGGTGT  Rev: TCATCAATCACGGACGTATCATC |
| *BRCA-2* | Fwd: TGCCTGAAAACCAGATGACTATC  Rev: AGGCCAGCAAACTTCCGTTTA |
| *RAD-51* | Fwd: CCTCCTCTTTAACGCCTCCTG  Rev: GGGGACAACTCCCAGACTTTTT |
| *CDK-2* | Fwd: CCAGGAGTTACTTCTATGCCTGA  Rev: TTCATCCAGGGGAGGTACAAC |
| *CDKN-1A* | Fwd: TGTCCGTCAGAACCCATGC  Rev: AAAGTCGAAGTTCCATCGCTC |
| *RAD-9A* | Fwd: CATTGACTCTTACATGATCGCCA  Rev: GCCAGGTGAAAGGGAAATGG |
| *MRE-11* | Fwd: GGGGCAGATGCACTTTGTG  Rev: GAAGCAAAACCGGACTAATGTCT |
| *ATR* | Fwd: GTTTTCCTCATCGAGGCATCTG  Rev: CCAAAATGACTGGTTAGCTGGTA |
| *BCL-2* | Fwd: TCGCCCTGTGGATGACTGA  Rev: CAGAGACAGCCAGGAGAAATCA |
| *PSA* | Fwd: CGGAGCAAACCTCGGAGTC  Rev: GCGGCCAGAAACAATGGATAG |
| *AR* | Fwd: GACGACCAGATGGCTGTCATT  Rev: GGGCGAAGTAGAGCATCCT |
| *C-MYC* | Fwd: CCTCCACTCGGAAGGACTATC  Rev: TGTTCGCCTCTTGACATTCTC |

**Table S2:** **List of PCa-specific oncomiRs expression primers**

| **Primers** | **Primer sequence (5’-3’)** |
| --- | --- |
| *miR-126* | Forward 5’- GTCGTATCCAGTGCAGGGTCCGAG -3’  Reverse 5’- GTATTCGCACTGGATACGAC -3’ |
| *miR-302* | Forward 5’-CCGCTCGAGAGTCTGTGGTTTAAATTCTGTCA -3’  Reverse 5’- CCGGAATTCGGTGGGCTCCCTTCAACT -3’ |
| *miR-16-5p* | Forward 5’- TAGCAGCACGTAAATATTGGCG -3’  Reverse 5’- TGCGTGTCGT­GGAGTC -3’ |
| *miR-205* | Forward 5’- TCCTTCATTCCACCGGAGTCTG -3’  Reverse 5’- GCGAGCACAGAATTAATACGAC -3’ |
| *miR-100-5p* | Forward 5’- GAACCCGTAGATCCGAACT -3’  Reverse 5’- CAGTGCGTGTCGTGGAGT -3’ |
| *miR-183-3p* | Forward 5’- CGCGCGTGAATTACCGAAG -3’  Reverse 5’- GTGCAGGG TCCGAGGT -3’ |
| *miR-183-5p* | Forward 5’- CGCGCTATGGCACTGGTAG -3’  Reverse 5’- GTGCAGGGTCCGAGGT -3’ |
| *miR-152-3p* | Forward 5’- GCAGTCAGTGCATGACAGA -3’  Reverse 5’- GTCCAGTTTTTTTTTTTTTTTCCAAG -3’ |
| *miR-152-5p* | Forward 5’- CAGAGGTTCTGTGATACACTC-3’  Reverse 5’- GGTCCAGTTTTTTTTTTTTTTTAGTC -3’ |
| *miR-146b* | Forward 5’- TGACCCATCCTGGGCCTCAA -3’  Reverse 5’- CCAGTGGGCAAGATGTGGGCC -3’ |
| *miR-200b-3p* | Forward 5’- TGCCTGGTAATGATGAGTCGT -3’  Reverse 5’- GTGTCGTGGAGTCGGCAATT -3’ |
| *miR-196a* | Forward 5’- CGTCAGAAGGAATGATGCACAG -3’  Reverse 5’- ACCTGCGTAGGTAGTTTCATGT -3’ |
| *miR-34a-5p* | Forward 5’- GCAGTGGCAGTGTCTTAG -3’  Reverse 5’- GTCCAGTTTTTTTTTTTTTTTACAAC -3’ |
| *miR-375* | Forward 5’- AGTTTGTTCGTTCGGCTC -3’  Reverse 5’- GGTCCAGTTTTTTTTTTTTTTTCAC -3’ |
| *miR-145-3p* | Forward 5’- GCCCTGTAGTGTTTCCTACTT -3’  Reverse 5’- GTGCAGGGTCCGAGGT -3’ |
| *miR-145-5p* | Forward 5’- GTCCAGTTTTCCCAGGAATCCCT -3’  Reverse 5’- TGGTGTCGTGGAGTCG -3’ |
| *miR-634* | Forward 5’- CAGTCTCAAACCAGCACC -3’  Reverse 5’- TATGGTTGTTCACGACTCCTTCAC -3’ |
| *miR-181* | Forward 5’- TGCGGAACATTCATTGCTGTC -3’  Reverse 5’- CCAGTGCAGGGTCCGAGGT -3’ |
| *miR-29b* | Forward 5’- GCGCGCTAGCACCATTTG -3’  Reverse 5’- CAGTGCAGGGTCCGAGGT -3’ |
| *miR-146a-3p* | Forward 5’- GCGAGGTCAAGTCACTAGTGGT -3’  Reverse 5’-CGAGAAGCTTGCATCACCAGAGAACG -3’ |
| *miR-21-5p* | Forward 5’- GCAGTAGCTTATCAGACTGATG -3’  Reverse 5’- GGTCCAGTTTTTTTTTTTTTTTCAAC -3’ |
| *miR-101* | Forward 5’- TGGGCTACAGTACTGTGATA -3’  Reverse 5’- TGCGTGTCGTGGAGTC -3’ |
| *miR-106a-5p* | Forward 5’- GATGCTCAAAAAGTGCTTACAGTGCA -3’  Reverse 5’- TATGGTTGTTCTGCTCTCTGTCTC -3’ |
| *miR-452* | Forward 5’- GCGAACTGTTTGCAGAGG -3’  Reverse 5’- CAGTGCGTGTCGTGGAGT -3’ |
| *miR-182-5p* | Forward 5’- ATCACTTTTGGCAATGGTAGAACT -3’  Reverse 5’- TATGGTTTTGACGACTGTGTGAT -3’ |
| *RNA-U6* | Forward 5’- ATTGGAACGATACAGAGAAGATT -3’  Reverse 5’- GGAACGCTTCACGAATTTG -3’ |

**Table S3: A.** Fold change of EMT-associated markers in the EBV-positive group as compared with the EBV-negative group. **B.** Fold change of PCa-associated genes in the EBV-positive group as compared with the EBV-negative group.

**A) Fold change of EMT-associated markers**

| EMT-associated genes | Fold Change (2−ΔΔCt) |
| --- | --- |
| *VIM (Vimentin)* | -1.12 |
| *CDH2 (N Cadherin)* | 1.3 |
| *CDH1 (E-cadherin)* | -1.2 |
| *SNAI2 (Slug)* | 2.1 |
| *SNAI1 (Snail)* | 2.1 |
| *TWIST1 (Twist)* | -1.2 |
| *ZEB1 (Zeb1)* | 1.0 |

**B) Fold change of PCa-associated genes**

| PCa carcinogenesis-associated genes | Fold Change (2−ΔΔCt) |
| --- | --- |
| *AR* | -1.02 |
| *PSA* | -1.5 |
| *BCL-2* | 1.1 |
| *CDK-2* | -2.1 |
| *MDM-2* | -1.2 |
| *c-Myc* | -1.9 |
| *CDKN-1A* | -1.4 |
| *CHEK-2* | 1.6 |
| *BRCA-1* | -1.25 |
| *CDC-20* | 1.07 |
| *MRE-11* | -2.27 |
| *CDKN-2A* | 1.6 |
| *RAD-9A* | -1.1 |
| *EP-300* | 1.02 |
| *AURK-B* | -1.8 |
| *CDKN-1B* | -1.15 |
| *BRCA-2* | -1.61 |
| *RAD-51* | -3.78 |
| *TP53* | -2.44 |
| *ATR* | -2.7 |
| *RB1* | -2.74 |

**Table S4: Calculated staining index (SI) based frequency distribution** EMT marker proteins (E-cadherin, vimentin, and N-cadherin) and androgen receptor in EBV positive and EBV negative PCa tissues.


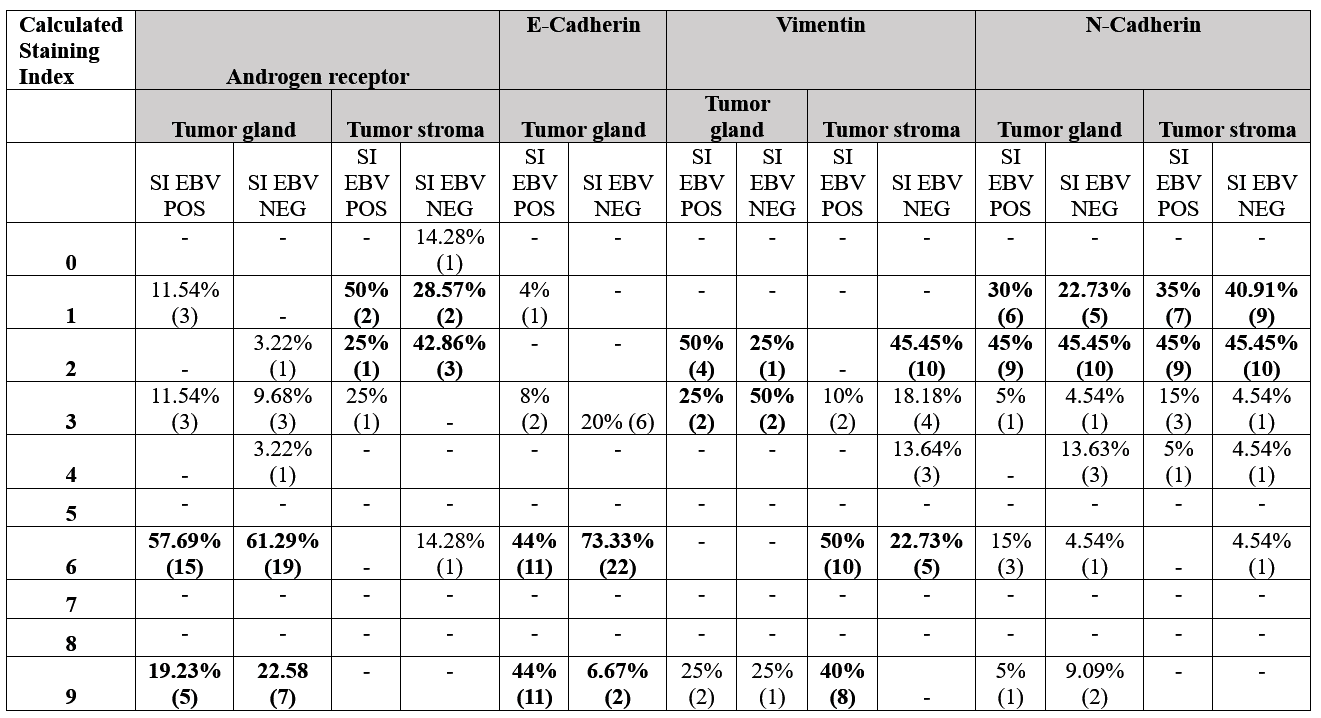


**Table S5: Immunohistochemistry-based expression analysis of AR and EMT markers in EBV-positive and EBV-negative PCa tissues**: it shows the descriptive statistics of staining intensity patterns and proportion of stained cells of Vimentin, E-Cadherin, N-Cadherin, and androgen receptor in EBV-positive and EBV-negative PCa tissues.

| **Staining Intensity (EMT markers and AR protein expression)** |  | **Tumor glands** | | **Tumor Stroma** | |
| --- | --- | --- | --- | --- | --- |
|  |  | EBV POS | EBV NEG | EBV POS | EBV NEG |
| **Vimentin** | 0 | 69.23% (18) | 87.88% (29) | 16% (4) | 31.25% (10) |
|  | 1+ | 0 % | 0% | 4% (1) | 43.75% (14) |
|  | 2+ | 15.38% (4) | 6.06% (2) | 12% (3) | 25% (8) |
|  | 3+ | 15.38% (4) | 6.06% (2) | 68% (17) | 0% |
| **N-Cadherin** | 0 | 28.57% (8) | 33.33% (11) | 28.57% (8) | 33.33% (11) |
|  | 1+ | 25% (7) | 24.24% (8) | 42.86% (12) | 45.45% (15) |
|  | 2+ | 35.71% (10) | 36.36% (12) | 21.43% (6) | 18.18% (6) |
|  | 3+ | 10.71% (3) | 6.06% (2) | 7.14% (2) | 3.03% (1) |
| **E-Cadherin** | 0 | - | - | - | - |
|  | 1+ | 10.71% (3) | 19.35% (6) | - | - |
|  | 2+ | 50% (14) | 74.19% (23) | - | - |
|  | 3+ | 39.28% (11) | 6.45% (2) | - | - |
| **Androgen receptor** | 0 | - | - | 83.33% (20) | 76% (19) |
|  | 1+ | 23.08% (6) | 12.90% (4) | 12.5% (3) | 16% (4) |
|  | 2+ | 53.85% (14) | 64.52% (20) | 4.17% (1) | 8% (2) |
|  | 3+ | 23.08% (6) | 22.58% (7) | - |  |

| **Proportion of cells stained (EMT markers and AR protein expression)** |  | **Tumor glands** | | **Tumor Stroma** | |
| --- | --- | --- | --- | --- | --- |
|  |  | EBV POS | EBV NEG | EBV POS | EBV NEG |
| **Vimentin** | 0 (0%) | 68% (17) | 87.88% (29) | 16% (4) | 31.25% (10) |
|  | 1 (1-10%) | 24% (6) | 9.091% (3) | 8% (2) | 43.75% (14) |
|  | 2 (11-50%) | 0% | 0% | 32% (8) | 25% (8) |
|  | 3 (>50%) | 8% (2) | 3.03% (1) | 44% (11) | 0% |
| **N-Cadherin** | 0 (0%) | 13.04% (3) | 40.62% (13) | 20% (5) | 40.62% (13) |
|  | 1 (1-10%) | 65.22% (15) | 28.12% (9) | 56% (14) | 34.37% (11) |
|  | 2 (11-50%) | 8.69% (2) | 18.75% (6) | 20% (5) | 21.87% (7) |
|  | 3 (>50%) | 13.04% (3) | 12.5% (4) | 4% (1) | 3.12% (1) |
| **E-Cadherin** | 0 (0%) |  |  | - | - |
|  | 1 (1-10%) | 3.57% (1) | 3.22% (1) | - | - |
|  | 2 (11-50%) | 3.57% (1) | 0% | - | - |
|  | 3 (>50%) | 92.86% (26) | 96.77% (30) | - | - |
| **Androgen receptor** | 0 (0%) | 3.70% (1) | 3.125% (1) | 84% (21) | 76% (19) |
|  | 1 (1-10%) | 11.11% (3) | 0% (0) | 12% (3) | 12% (3) |
|  | 2 (11-50%) | 3.70% (1) | 6.25% (2) | 0% | 12% (3) |
|  | 3 (>50%) | 81.48% (22) | 90.62% (29) | 4% (1) | 0% |


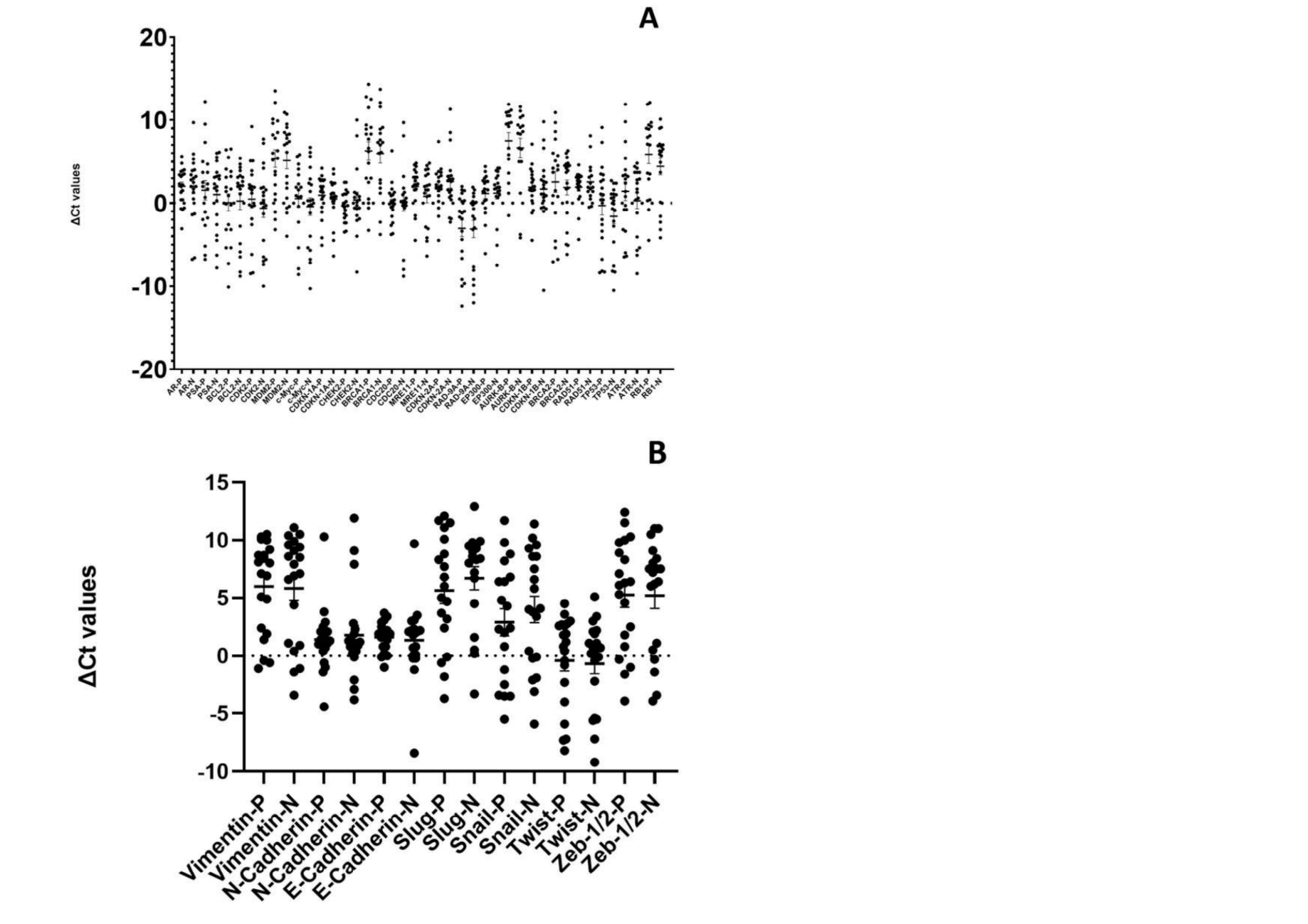


**Supplementary Figure S1: Differential expression of genes associated with EMT and Prostate oncogenesis A) Expression of EMT-associated genes in EBV-positive and EBV-negative PCa samples:** The scatter plot shows ∆CT values of EMT-associated markers (*E-cadherin, N-cadherin, vimentin, Slug, Snail, Twist,* and *Zeb1/2*) in EBV-positive and EBV-negative PCa samples. Letter P and N at the end of the genes denote EBV-positive and EBV-negative PCa samples, respectively. The error bars indicate SEM. **B)** **Expression of prostate carcinoma-associated genes in EBV-positive and EBV-negative PCa samples:** The scatter plot shows ∆CT values of PCa-associated genes (*c-MYC, AURKB, CDC20, EP300, MDM2, TP53, RB1, CHEK2, CDKN-1B, CDKN2A, BRCA1, BRCA2, RAD51, CDK2, CDKN1A, RAD9A, MRE11, ATR, BCL2, PSA,* and *AR*) in EBV-positive and EBV-negative PCa tissues was determined using comparative Ct method (mean with SEM). Letter P and N at the end of the genes denote EBV-positive and EBV-negative PCa samples, respectively. The error bars indicate SEM.


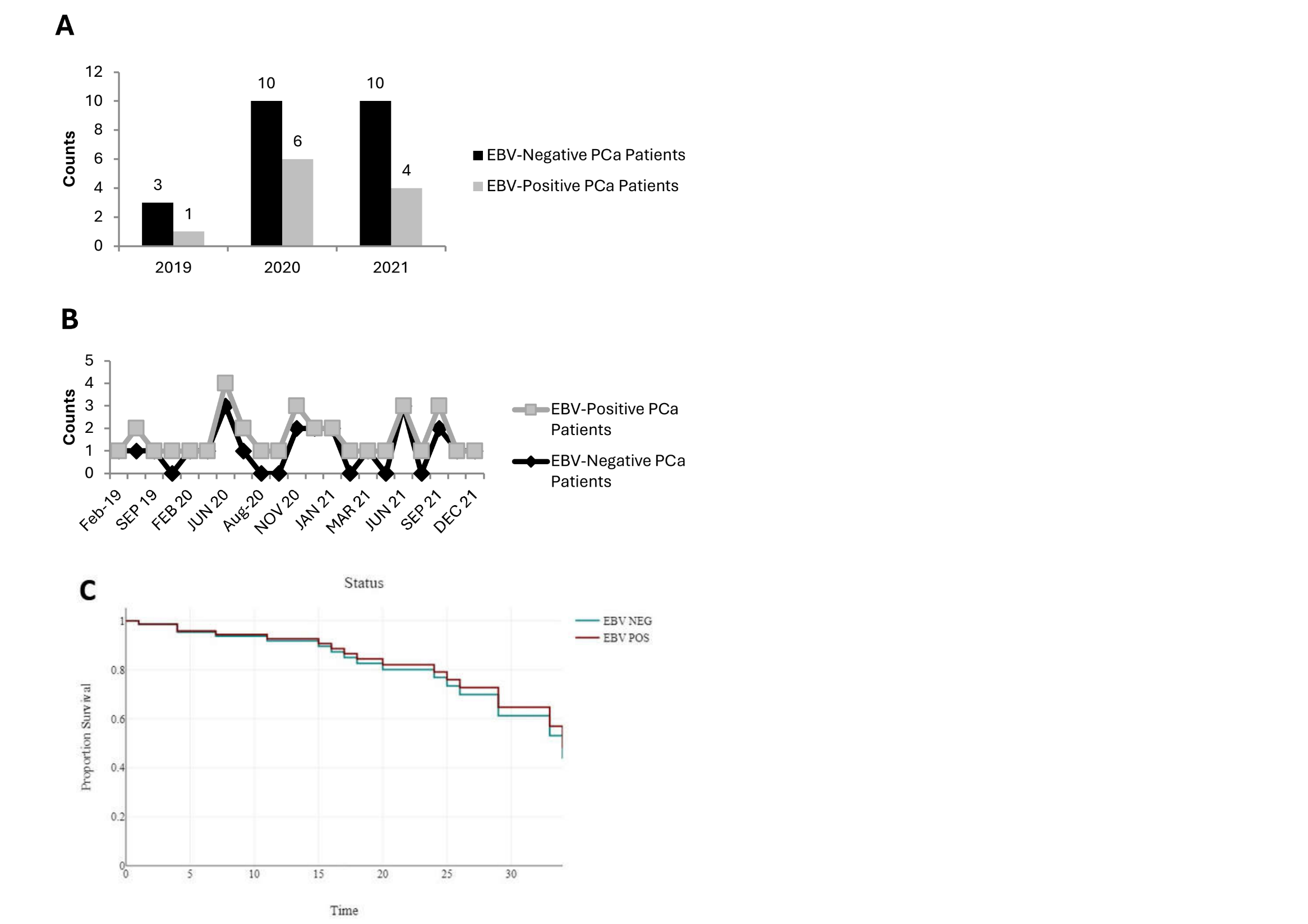


**Figure S2: Year-wise and month-wise distribution of deaths in EBV-positive and -negative PCa patients:**  The figure shows **A)** year-wise, **B)** month-wise number of deaths (count) in EBV-positive and -negative PCa patients. **C)** Proportion Survival versus time scale in months in EBV-positive versus EBV-negative PCa patient samples.
